# Lessons Learned From a Peer-Supported Differentiated Care and Nutritional Supplementation for People With TB in a Southern Indian State

**DOI:** 10.9745/GHSP-D-23-00504

**Published:** 2024-08-27

**Authors:** Hemant Deepak Shewade, A. James Jeyakumar Jaisingh, Prabhadevi Ravichandran, S. Kiran Pradeep, Sripriya Pandurangan, Subrat Mohanty, T. Daniel Rajasekar, R. Vijayaprabha, G. Kiruthika, K.V. Suma, Delphina Peter Pathinathan, Deiveegan Chidambaram, K. Sivagami, Anupama Srinivasan, Reuben Swamickan, Amrita Goswami, D. Sivaranjani, Ramya Ananthakrishnan, Asha Frederick, Manoj V. Murhekar

**Affiliations:** a ICMR–National Institute of Epidemiology (ICMR-NIE), Chennai, India.; b REACH (Resource Group for Education and Advocacy for Community Health), Chennai, India.; c Office of the World Health Organization (WHO) Representative to India, WHO Country Office, New Delhi, India.; d U.S. Agency for International Development, New Delhi, India.; e TB Free Tamil Nadu Survivors led Network, Krishnagiri, India.; f State TB Cell, Government of Tamil Nadu, Chennai, India.

## Abstract

Two critical components of patient support systems for people with TB are regular counseling and locally managed nutritional support. As part of an ongoing differentiated TB care initiative called Tamil Nadu Kasanoi Erappila Thittam (meaning TB death-free initiative in Tamil, TN-KET) to reduce TB deaths, adults with TB with very severe undernutrition, respiratory insufficiency, or poor performance status are identified at diagnosis (triage-positive) and prioritized for referral, comprehensive clinical assessment, and inpatient care. Between January and June 2023, in 6 districts, a pilot exercise was conducted in which trained TB survivors, known as TB champions, provided baseline counseling and additional counseling (if required) to triage-positive people with TB at diagnosis. Additionally, people with TB with severe undernutrition were prioritized for nutritional supplementation for at least 3 months. Among 652 people with TB who were triage-positive at diagnosis, the program staff shared details of 145 (22%), and all were counseled by TB champions (baseline counseling). Program staff identified 74 (11%) triage-positive people with TB who required additional counseling (i.e., those refusing referral or admission or continued admission), and 71 (96%) were counseled by TB champions. Among these, 54 (76%) were admitted or readmitted and successfully discharged. In addition, among 1,042 people with TB with severe undernutrition, program staff shared details of 390 (38%), of which 60% received nutritional supplementation through the efforts of TB champions. We conclude that TB champions were able to provide quality and timely peer support through direct counseling and by mobilizing local resources for nutritional support. The engagement of TB champions can be further strengthened by establishing robust coordination mechanisms with the TB program. Lessons from this pilot will contribute to the Tamil Nadu State TB Cell’s plans to expand the role of TB champions and enhance community participation to end TB in India.

## Abstract (precis)

This pilot from southern India highlights the potential role of trained TB champions in counseling severely ill people with TB and facilitating targeted nutritional supplementation by mobilizing local resources.

## Teaser

**Key Findings:**

- TB champions—individuals who have been affected by TB, have completed treatment, and subsequently been trained to provide support and counseling—provided baseline and additional counseling for more than 95% of severely ill people with TB and facilitated nutritional supplementation for 60% of those with severe undernutrition.
- The unique ability of TB champions to draw on their personal experiences of TB to motivate others is evident.

**Key Implications:**

- Strong coordination must be established between the TB program and TB champions to maximize their participation.
- Policymakers can learn from TB champions’ experiences regarding enablers and barriers to implementation for policy adaptation.

## INTRODUCTION

In Tamil Nadu, a southern Indian state with a population of about 72 million, 322 persons per 100,000 population (2019–2021) are estimated to have TB, making it one of the high-burden states in India.^1^ Due to the COVID-19 pandemic, the reported TB death rate in Tamil Nadu increased to 6.4%, and more than 70% of deaths were within 2 months (early deaths).^2^

India’s National Strategic Plan for TB Elimination (2017–2025) proposes a community-based and patient-centric approach to achieve the 2030 Sustainable Development Goals TB targets by 2025.^3^ Two of the 9 patient support system components in the plan include (1) initial and frequent follow-up counseling for the person with TB and their family and (2) locally managed supplementary nutritional support, in addition to financial aid in the form of direct benefit transfer of INR500 per month through the Ni-kshay Poshan Yojana.^4^ The first step in achieving this is to involve and empower TB champions in routine TB program settings.^3^ The national strategic plan recommends that there must be at least 2 TB champions in every village in India. With an average population of 2,000 per village, 1,300,000 TB champions will be required.^5^

A TB champion is a person who has been affected by TB and has completed the treatment and subsequently been trained using the National TB Elimination Program’s (NTEP) standard “TB survivor to TB champion” curriculum.^5^ Trained TB champions draw on their personal lived experiences of TB to act as a link between people with TB and the TB program. In addition to providing considerable knowledge on TB for treatment literacy, they also provide emotional support, linkages to care, treatment adherence support, stigma reduction support, and practical assistance, as well as link those affected with resources, benefits, schemes, and opportunities.^5^ They also take ownership and participate in community engagement activities like active case-finding, observing World TB day, and sensitizing Panchayat raj (local government) institutions and village health nutrition and sanitation committees. There could be an overlap of roles with existing community health workers. However, the TB champions’ focused approach toward TB and lived experience can effectively motivate TB patients by narrating their personal journey.

TB champions can provide emotional support, linkages to care, treatment adherence support, stigma reduction support, and practical assistance to TB patients.

### Tamil Nadu Kasanoi Erappila Thittam Initiative

Since April 2022, Tamil Nadu has been implementing India’s first statewide differentiated TB care initiative called Tamil Nadu Kasanoi Erappila Thittam (meaning TB death-free initiative in Tamil, TN-KET) to reduce TB deaths. The differentiated TB care initiative aims to identify severely ill people with TB through triaging at diagnosis and provide inpatient care to triage-positive people.^6^ Planned and jointly led by the Tamil Nadu State TB cell and ICMR-National Institute of Epidemiology (ICMR-NIE), TN-KET is being implemented as a routine activity under the TB program in the state using the existing health system workforce (not externally funded).^6^

Under TN-KET, in 30 NTEP districts of Tamil Nadu (excluding Chennai, the capital), adults (aged older than 15 years) with TB (not known to be drug resistant at diagnosis) notified from public facilities are systematically triaged. The indicators in the triage tool are (1) body mass index (BMI) of 14 kg/m^2^ or less, (2) BMI of 14.1–16 kg/m^2^ with pedal edema, (3) respiratory rate of more than 24 breaths/minute, (4) oxygen saturation of less than 94%, and (5) unable to stand without support. Those with very severe undernutrition (indicator 1 or 2) or respiratory insufficiency (indicator 3 or 4), or poor performance status (indicator 5) at diagnosis are documented as triage-positive and prioritized for referral to subdistrict, district, or teaching hospitals, followed by comprehensive clinical assessment and inpatient care.^6^^,^^7^

Monthly reports are generated based on the data transcribed into the severe TB web application. The process indicators developed at the state and district level are as follows: (1) percentage triaged at diagnosis; (2) percentage triage-positive who are referred, comprehensively assessed, and confirmed as severely ill; (3) percentage confirmed severely ill who are admitted; and (4) median duration of admission.

### Accountability Leadership by Local Communities for Inclusive, Enabling Services Project

Through the Accountability Leadership by Local Communities for Inclusive, Enabling Services (ALLIES) project, TB champions, as survivors and role models, provide valuable support to those with TB and their families.^8^^,^^9^ Supported by the U.S. Agency for International Development, the 5-year ALLIES project (October 2019 to September 2024) strives to create an enabling environment toward ending TB through community-led action.^9^ The project is being implemented by the Resource Group for Education and Advocacy for Community Health **(**REACH), a nonprofit organization based in India working on TB,^10^ in 3 districts each in Chhattisgarh, Jharkhand, and Odisha and 6 districts in Tamil Nadu (Vellore, Villupuram, Tiruchirappalli, Coimbatore, Cuddalore, and Krishnagiri).^9^

These trained TB champions provide a comprehensive package of psychosocial care to people with TB in their districts, including peer counseling, treatment literacy, family counseling, and support with stigma and discrimination. In addition, they advocate for greater involvement of local stakeholders and decision-makers (elected representatives, community-based organizations, local industries, and media) in the TB response, mobilize local resources, and educate communities about TB. ALLIES district strategists appointed at these 6 districts of Tamil Nadu coordinate the smooth functioning of activities.

## PEER SUPPORT AND NUTRITIONAL SUPPLEMENTATION PILOT EXERCISE

Between January and June 2023, as a pilot exercise, TN-KET collaborated with the ALLIES project to ensure triage-positive people with TB at diagnosis received counseling by TB champions. The baseline counseling (residence or hospital, in person or over the phone) to triage-positive at diagnosis was provided in addition to the routine counseling and information provided by district NTEP staff to all people with TB. As part of baseline counseling, TB champions counseled triage-positive people with TB to begin or continue treatment, advised family members of the care and support required, and provided guidance on the importance of nutrition during TB treatment. TB champions drew on their own personal experiences of TB, sharing how they were impacted by the disease and subsequently recovered. They served as friends and counselors, clarifying any misconceptions that people with TB and their families had. They also facilitated local ambulance services for the severely ill and provided continued support through phone follow-ups.

TB champions served as friends and counselors, clarifying any misconceptions that people with TB and their families had.

They supported those not completing the TN-KET care cascade by providing additional counseling to ensure they completed the admission process and were successfully discharged for ambulatory directly observed treatment. Some common reasons for people with TB refusing to get admitted included the unavailability of a family member to accompany them (mandatory for admissions), fear they would lose income during the admission period, and reluctance to get admitted to public health facilities. TB champions provided counseling that addressed these specific concerns.

There exists a bidirectional solid causal link between TB and undernutrition.^11^ The World Health Organization recommends that all persons with active TB receive appropriate counseling based on their nutritional status at diagnosis and throughout treatment.^12^ Hence, we intended to prioritize nutritional supplementation (covering around one-third of daily calorie and protein requirement) for at least 3 months among people with TB with severe undernutrition (BMI 16 kg/m^2^ or less) at diagnosis. The district strategist and TB champion visited the patient’s home to assess their health and family condition. If nutritional supplementation was needed, potential donors were identified with the help of TB champions. Donors included local *panchayat* leaders, local philanthropists, industries, and nongovernmental organizations. They collected the nutritional supplementation (dry rations) from the donors and distributed it at the patient’s home in the first month, followed by health facility-based distribution and nutritional counseling by the NTEP team.

In the 6 districts, 24 TB champions (district-wise range: 3–5, 22 female and 2 male) received a fixed monthly honorarium from the project. Additionally, they were given an incentive of INR300 for every successful admission of a severely ill TB patient at the TN-KET nodal facilities and INR200 for every home-based counseling, mobilization of resources for nutrition support, and distribution.

## RESULTS

Between January and June 2023, in the 6 districts, 652 people with TB were triage-positive. Of this cohort, details of 145 people (22%, district-wise range: 10%–45%) were shared by NTEP with TB champions for baseline counseling. Additional counseling was requested for 74 (11%) triage-positive people with TB. This included those who refused admission (n=64) and those who left the health facility against medical advice (n=10). The details were shared through a copy of a paper-based triage tool that had no personal identifiers other than the unique identifier used in NTEP (Ni-kshay ID), date, and facility of diagnosis. All 145 (100%) were provided baseline counseling by TB champions (37% female). Additional counseling was provided to 71 (96%) people (28% female). Of these, 54 (76%) were successfully admitted to the health facility and subsequently discharged (one-third were female).

There were 1,042 people with TB with severe undernutrition. Of this cohort, details of 390 people (38%, district-wise range: 19%–100%) were shared by NTEP with TB champions. Of these 390, they facilitated nutritional supplementation for at least 3 months for 238 (61%, district-wise range: 20%–93%). The results are summarized in the Figure.

**FIGURE:**
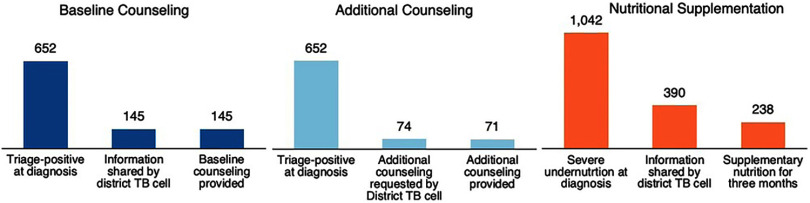
Number of People Who Received Baseline Counseling, Additional Counseling, and Nutritional Supplementation From TB Champions in Six Districts of Tamil Nadu, India, January to June 2023

In 1 district, resource mobilization was done for therapeutic nutrition for triage-positive admitted with very severe undernutrition. Raw materials for formula 75 (F-75: puffed rice, skimmed milk powder, sugar, vegetable oil) or F-75 premix tins were provided to hospitals. Ten admitted patients benefited from this.

## DISCUSSION

We observed that TB champions played an important role in motivating people with TB for admission or readmission. Almost all triage-positive people with TB referred to TB champions received baseline and additional counseling. Of those severely ill TB patients who were not willing to be admitted even after various attempts by the health staff, 76% were successfully admitted and supported during their course of treatment (through additional counseling by TB champions). Based on our program experience, we speculate that these patients would not have possibly been admitted in the absence of counseling from TB champions. Six in 10 patients with severe undernutrition at diagnosis received nutritional supplementation for 3 months. The proportion receiving nutrition supplementation was an underestimate as we used all with severe undernutrition as the denominator while supplementation was provided only to those after completing a need assessment exercise by the ALLIES team.

TB champions are highly motivated to work with people with TB and have the capacity to catalyze change in their communities.^5^ Some field-level challenges included limited sharing of information with TB champions in real time. A potential reason could be that there was an element of selection of those most needing support by NTEP staff. Further qualitative exploration is required among NTEP program managers and staff to understand their perspectives. By addressing these, we may be able to maximize the benefits to people with TB.

TB champions are highly motivated to work with people with TB and have the capacity to catalyze change in their communities.

A qualitative study in Kerala (neighboring state of Tamil Nadu), which analyzed TB support services in at least 14 districts, found that these services improved early diagnosis, good adherence to treatment, reduced patient inconveniences, stigma reduction, prevention of out-of-pocket expenditure, and emotional support.^13^ Our experience adds to the existing published literature where TB champions provided targeted support in 2 specific patient support mechanisms: (1) ensuring severely ill people with TB at diagnosis get counseled and complete their admission and (2) nutritional supplementation among those with severe undernutrition at diagnosis. We followed a targeted approach considering the number of TB champions that were available for the pilot.

The 24 TB champions over 6 districts are much less than the recommendation of 2 TB champions per village. While NTEP plans to have 2 TB champions in every village in the long term, the current pilot project used the existing TB champions working in the same geographical setting. This was not based on a calculation of TB champions to population ratio. We are not suggesting that this density of TB champions in our pilot is adequate. In addition, through this pilot initiative, we focused only on triage-positive people, which is 10%–15% of all people with TB (at diagnosis) in a district.^14^ Differentiated TB care is one of the many activities under the TB program, and the TB champions provided care to severely ill and severely undernourished patients, who were a small proportion of all TB patients. Scale-up in line with the national recommendation to cover all communities, TB patients, and all services (beyond differentiated TB care) can only be done by the state and is beyond the scope of the project.

As the Tamil Nadu state TB cell plans to expand the engagement of TB champions across the state, lessons from this partnership will immensely help and contribute to the ambitious aim of ending TB in India. Policymakers can learn from TB champions’ experiences regarding enablers and barriers in implementation for policy adaptation. Pilots like these contribute to this process. This will also pave the way for the scalability and sustainability of the initiative and assessment of changes in TB death rates. The role of TB champions in mobilizing local resources and facilitating supplementary nutrition is critical, particularly for people with TB who are severely undernourished. Trained TB champions are ideally placed to provide peer support and improve treatment literacy, which are significant factors in motivating people with TB to continue and complete treatment successfully.

## Notes

**Peer Reviewed**

**First Published Online:** July 18, 2024.
